# Inter-Subject Variability in Human Atrial Action Potential in Sinus Rhythm versus Chronic Atrial Fibrillation

**DOI:** 10.1371/journal.pone.0105897

**Published:** 2014-08-26

**Authors:** Carlos Sánchez, Alfonso Bueno-Orovio, Erich Wettwer, Simone Loose, Jana Simon, Ursula Ravens, Esther Pueyo, Blanca Rodriguez

**Affiliations:** 1 Biosignal Interpretation and Computational Simulation (BSICoS), Aragón Institute of Engineering Research (I3A) and Aragón Health Research Institute (IIS), University of Zaragoza, Zaragoza, Spain; 2 Biomedical Research Networking Center in Bioengineering, Biomaterials and Nanomedicine (CIBER-BBN), Zaragoza, Spain; 3 Department of Computer Science, University of Oxford, Oxford, United Kingdom; 4 Department of Pharmacology and Toxicology, Dresden University of Technology, Dresden, Germany; Gent University, Belgium

## Abstract

**Aims:**

Human atrial electrophysiology exhibits high inter-subject variability in both sinus rhythm (SR) and chronic atrial fibrillation (cAF) patients. Variability is however rarely investigated in experimental and theoretical electrophysiological studies, thus hampering the understanding of its underlying causes but also its implications in explaining differences in the response to disease and treatment. In our study, we aim at investigating the ability of populations of human atrial cell models to capture the inter-subject variability in action potential (AP) recorded in 363 patients both under SR and cAF conditions.

**Methods and Results:**

Human AP recordings in atrial trabeculae (n = 469) from SR and cAF patients were used to calibrate populations of computational SR and cAF atrial AP models. Three populations of over 2000 sampled models were generated, based on three different human atrial AP models. Experimental calibration selected populations of AP models yielding AP with morphology and duration in range with experimental recordings. Populations using the three original models can mimic variability in experimental AP in both SR and cAF, with median conductance values in SR for most ionic currents deviating less than 30% from their original peak values. All cAF populations show similar variations in G_K1_, G_Kur_ and G_to,_ consistent with AF-related remodeling as reported in experiments. In all SR and cAF model populations, inter-subject variability in I_K1_ and I_NaK_ underlies variability in APD_90_, variability in I_Kur_, I_CaL_ and I_NaK_ modulates variability in APD_50_ and combined variability in I_to_ and I_Kur_ determines variability in APD_20_. The large variability in human atrial AP triangulation is mostly determined by I_K1_ and either I_NaK_ or I_NaCa_ depending on the model.

**Conclusion:**

Experimentally-calibrated human atrial AP models populations mimic AP variability in SR and cAF patient recordings, and identify potential ionic determinants of inter-subject variability in human atrial AP duration and morphology in SR versus cAF.

## Introduction

Atrial arrhythmias constitute a huge burden to health-care systems in the developed countries, both because of their high rate of incidence and also because they usually lead to other deadly cardiovascular diseases such as stroke [1]. A large body of research has aimed at characterizing human atrial membrane kinetics and their implication in cellular and tissue electrophysiology, in an attempt to improve our understanding of the initiation and maintenance of atrial arrhythmias [2]. An important challenge in understanding human atrial electrophysiology is the large inter-subject variability present in electrophysiological recordings obtained from human samples. Characterizing and understanding inter-subject variability in atrial cellular electrophysiology is important to determine not only the physiological range of action potential (AP) properties, but also differences under disease conditions and in the response to treatment between patients. The causes of electrophysiological variability are largely unknown. However, recent studies highlight its temporally-dynamic nature and a variety of causes, pointing towards genetic differences (including sex [3]) but also factors such as age, circadian rhythms [4] and long term drug effects [5].

In spite of its potential importance, variability is often ignored in both experimental and theoretical studies of atrial electrophysiology properties, probably due to its complexity.

Experimental studies often focus on average values obtained in a limited number of recordings. This is reflected in theoretical studies, and mathematical models of human atrial electrophysiology reported in the literature are all based on a single set of parameters. The human atrial models therefore yield a single AP, which exhibits significant differences in shape and duration for different models under similar simulated conditions [6]. The differences in outcomes obtained using different human atrial models may in fact reflect the large degree of variability in the data that are used in their construction.

Recently, inter-subject variability in electrophysiological properties has been investigated in a number of studies, which have mostly focused on ventricular rather than atrial electrophysiology [7]–[10]. Previous studies include, amongst others, investigation of the ionic basis of variability in human ventricular APs [11], in canine ventricular AP for drug safety assessment [12], in rabbit Purkinje cellular electrophysiology for investigation of variability in the response to drug action [13] and in rabbit ventricular electrophysiology at different pacing rates [14]. Furthermore, inter-subject variability has also been previously investigated in studies with other cell types such as neuronal electrical activity in crabs for either the study of variability in channel expressions [15], or the correlation between channel expressions [16], and variability in human ventricular APs in heart failure [11].

As a way to incorporate experimentally-reported variability in mathematical models of biological systems, a population of models approach has been recently proposed for cellular electrophysiology [8], [11], [13], [14], [17], [18]. The population of models approach aims to provide a new framework to overcome an important limitation of current models imposed by the implicit assumption that all cells of a certain type have quantitatively identical ionic properties. Simulation studies using populations of models allow exploring potential causes and implications of the cell level variability exhibited in experimental recordings, and thus aim at facilitating the generation of new hypothesis on likely mechanisms of variability that are otherwise very opaque using experimental techniques alone.

In this study, we investigate the ability of populations of human atrial AP models based on three recently published models to capture the inter-subject variability in human atrial AP, as exhibited in recordings from over 350 atrial trabeculae from sinus rhythm (SR) and chronic AF (cAF) patients. The human atria models within each population (based on one of the three models) share the same equations but include different combinations of sampled ionic current conductance values, as previously described [8], [9], [11], [13], [19]. The experimentally-calibrated human atrial AP model populations are then used to quantify the contribution of specific ionic currents to determining inter-subject variability in cellular human atrial AP duration (APD) and morphology in the populations, and to determine potential differences between the populations based on SR and cAF patients’ recordings. Three different baseline AP models are used in our study to construct the populations in both SR and cAF. This is done in order to consider differences in the structure of the models equations as well as in parameters in our investigations, and also to evaluate the model independence of the identified mechanisms of variability.

## Methods

### Experimental Dataset

The experimental datasets were obtained in studies with human samples conforming to the Declaration of Helsinki. The study was approved by the Ethics Committee of Dresden University of Technology (No. EK790799). Each patient gave written, informed consent. Right atrial appendages were obtained from 363 patients with SR (214 patients) and with cAF (149 patients) undergoing cardiac surgery for coronary artery bypass grafting or mitral/aortic valve replacement. SR patients may include atrial tissue from AF patients that have had paroxysmal and recent onset AF. Antiarrhythmic drugs were discontinued before the study.

APs were recorded with standard intracellular microelectrodes in atrial trabeculae (469 recordings from 363 patients: SR, n = 254, from 214 patients; cAF, n = 215, from 149 patients) [20], [21]. Bath solution contained (in mM): NaCl 127, KCl 4.5, MgCl_2_ 1.5, CaCl_2_ 1.8, glucose 10, NaHCO_3_ 22, NaH_2_PO_4_ 0.42, equilibrated with O_2_-CO_2_ [95∶5] at 36.5±0.58°C, pH 7.4. Preparations were regularly stimulated at 1 Hz for at least 1 h before data acquisition [20], [21]. Human myocytes were isolated enzymatically from atrial appendages as previously described [22]. The following parameters were quantified to characterize inter-subject variability in human atrial AP: APD at 20, 50, and 90% repolarization (APD_20_, APD_50_, APD_90_, respectively), AP amplitude (APA), resting membrane potential (RMP), plateau potential defined as the potential measured at 20% of the APD_90_ time (V_20_), and maximum upstroke velocity (dV/dt_max_). Minimum, maximum and mean±standard deviation values for these biomarkers are presented in Table 1.

**Table 1:** Human atrial AP biomarkers’ ranges in SR and cAF patients.

	SR			cAF		
	*Minimum Value*	*Maximum Value*	*Mean±SD*	*Minimum Value*	*Maximum Value*	*Mean±SD*
APD_90_ (ms)	190	440	318±42	140	330	216±35***
APD_50_ (ms)	6	200	139±44	30	180	102±28***
APD_20_ (ms)	1	60	7±8	1	75	30±18***
APA (mV)	75	120	95±7	80	130	102±8***
RMP (mV)	−85	−65	−74±4	−85	−65	−77±4***
V_20_ (mV)	−35	10	−16±6	−30	20	−4±11***
dV/dt_max_ (V/s)	40	420	220±68	40	420	232±70*

(Statistical significance between SR and cAF: *p<0.05; **p<0.01; ***p<0.001).

### Human Atrial Electrophysiology Cell Models

Three recent human atrial AP models were used as a base to construct the computational AP model populations, the Maleckar et al. [23], the Courtemanche et al. [24], and the Grandi et al. [25] models. For simplicity, these models will be referred to in the text by their first authors’ names.

All three models provide biophysically-detailed descriptions of human atrial cellular electrophysiology including main transmembrane ionic currents, including the fast sodium current I_Na_, the L-type calcium current I_CaL_, the transient outward potassium current I_to_, the ultra-rapid potassium current I_Kur_, the inward rectifier potassium current I_K1_, the rapid and slow components of the delayed rectifier potassium current (I_Kr_ and I_Ks_) and those associated with the sodium/potassium pump (I_NaK_) and the sodium/calcium exchanger (I_NaCa_). The models also include representation of the intracellular calcium handling and ionic homeostasis regulating sodium, potassium and calcium intracellular concentrations, which also determine the time course of the human atrial AP. Human atrial cell models have been reviewed in detail in previous publications [6], [26]–[28] and here we provide a brief description on their main characteristics.

The Maleckar model is a modified version of the original human atrial model by Nygren et al. [29], which includes a reformulation of I_Kur_ and I_to_ to better reproduce rate dependent properties. The calcium subsystem is based on the rabbit atrial model by Lindblad et al. [30]. The Maleckar model yields a simulated AP with a triangular shape, and a calcium transient with large peak and rapid decay. The Courtemanche model is one of the first human atrial cell models, published in 1998, the same year the Nygren model was also published. Both models include representation of the 12 main transmembrane ionic currents, largely based on the same human atrial model but with significant model differences. In the Courtemanche model for example, the calcium dynamics are based on the Luo-Rudy dynamic AP model [31]. Thus, the simulated AP using the Courtemanche model is characterized by a longer plateau phase and less triangular shape compared to the Nygren and Maleckar models, and also a longer-lasting calcium transient with a rapid upstroke but slow decay. Finally, the Grandi model was developed largely based on their previous human ventricular model [32], and therefore the formulation of transmembrane currents and calcium dynamics differs significantly from the Maleckar and Courtemanche models. In addition to the main transmembrane currents, the Grandi atrial model also includes the formulation of two chloride and a potassium plateau current. The calcium subsystem model is based on the one in the rabbit ventricular model by Shannon et al. [33]. The AP morphology is triangular with a longer APD than the one obtained with the other models, and the calcium transient displays a slow rise but low amplitude and slow decay.

### Populations of Models of Human Atrial Electrophysiology

To capture inter-subject variability, three populations of sampled models of human atrial electrophysiology for both SR and cAF were generated, each one based on one of the original AP models (i.e. Maleckar, Courtemanche and Grandi models). All models in each population shared the same equations but the most important ionic current conductances in determining the human atrial AP were varied with respect to their original values [34]. According to our previous sensitivity analysis study [34], the most relevant parameters were the conductances of I_K1_, I_CaL_, I_to_, I_Kur_, and maximal sodium/potassium pump (I_NaK_) and sodium/calcium exchanger currents (I_NaCa_) [34]. Most other ionic current conductances and gating variable kinetics exhibit small or negligible effects on the investigated AP biomarkers.

Our first step is to estimate the median values and range of variation for the six key ionic conductances required to obtain simulated APs to be within experimental range for each of the models. To do so and to minimize computational expense, we first constructed coarse model populations with 2275 different ionic conductance combinations sampled over a ±100% variation range around their values in the original models, using the methodology described in [35]. The method works by varying different parameters at different frequencies, encoding the identity of parameters in the frequency of their variation. Briefly, the sampling methodology consists of first generating sinusoidal functions with particular frequencies with N_S_ samples for each ionic conductance (i.e. a search curve). The frequencies assigned to the parameters must meet several criteria to avoid aliasing and interference effects; see [35] for a detailed discussion of how frequencies are chosen. Then search curves were randomly resampled N_R_ times to avoid repetition of values due to the periodicity properties of sinusoidal functions. The total number of models for each population, 2275, was obtained by multiplying N_S_ ( = 65), N_R_ ( = 5) and the number of ionic conductances ( = 6) plus an additional “dummy” (free) parameter ( = 6+1). The free parameter is used as a security step to guarantee the statistical significance of the other parameters (i.e., the ionic conductances) to round-off artifacts in the global method as described previously [35]. We ensured that the 2275 combinations were consistent with the sampling methodology without losing sampling resolution [35]. Since we used three different original AP models, the total number of unrestricted combinations of parameters was 6825.

**Figure 1:**
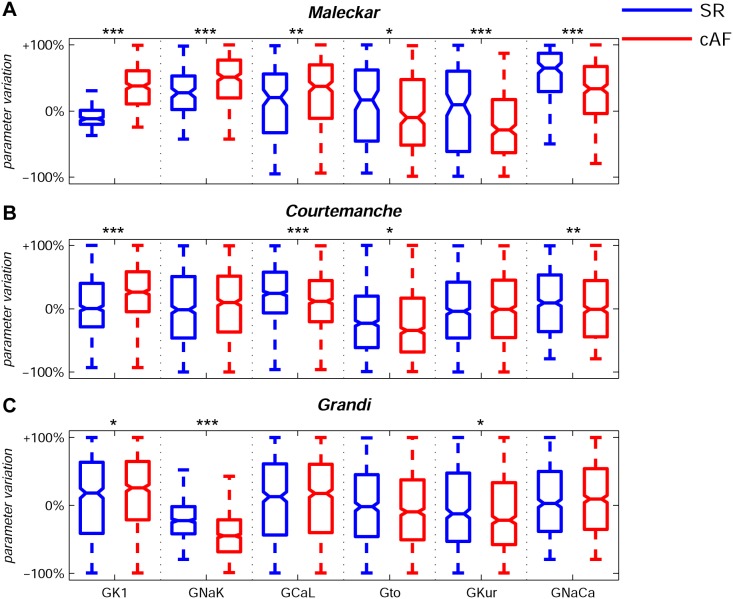
Ranges of variability of ionic conductances in the human atrial AP model populations. Median values and ranges of variability of ionic conductances G_K1_, G_NaK_, G_CaL_, G_to_, G_Kur_ and G_NaCa_ in the experimentally-calibrated populations of human atrial AP models, sampled within a ±100% range of their original values in the Maleckar (**A**), Courtemanche (**B**) and Grandi (**C**) human atria AP models in SR (blue) and cAF (red). Each boxplot represents the range covered by the ionic conductances: the edges of the box are the 1^st^ and 3^rd^ quartiles, the whiskers extend to the most extreme datapoints, the estimated median physiological value is the central horizontal line and the notch around the median is the 5% significance level. (Mann-Whitney U test: *p<0.05; **p<0.01; ***p<0.001).

The Maleckar, Courtemanche and Grandi default models were initially preconditioned by pacing at 1 Hz (using a 2 ms stimulus duration, twice diastolic threshold amplitude) until the steady-state was reached (changes in state variables between consecutive stimuli measured at the end of each cardiac cycle smaller than 1%). All AP models within the populations were paced at 1 Hz, and APs were analyzed following a train of 90 periodic stimuli to quantify APD_20_, APD_50_, APD_90_, APA, RMP, V_20_ and dV/dt_max_ for each model in each of the populations. AP models were selected as in physiological range if all AP properties were in the experimental ranges described in Table 1 for SR and cAF, respectively. Models with AP properties out of the experimental ranges described in Table 1 were discarded from the populations. Additionally, models presenting abnormalities such as delayed afterdepolarizations (DADs) were also removed for subsequent analysis. Figure 1 shows median physiological values for each of the six ionic conductances from these initial populations calibrated with the human atria recordings for SR and cAF patients. Note that most median values deviate from the original model value (represented as 0% in Figure 1), indicating that the original models are not representative of median behavior in our experimental recordings and that the calibration step is required. The deviation of the median value from the original one is often moderate for all ionic currents and all models, and within a ±30% range in SR, with the exception of the sodium/calcium exchanger in the Maleckar model which requires a significant up-regulation in SR (+65%) for models to be within physiological range, as shown in Table 2. The ranges obtained for each of the conductances vary significantly between ionic currents, indicating differences in the sensitivity of the AP to variation in each of the currents. The ionic ranges in SR and cAF often overlap, reflecting that there is also overlap in the AP biomarkers distributions. Importantly, a comparison between SR and cAF shows an overall increase in G_K1_ and a decrease in G_to_ and G_Kur_ in cAF using the three models (except for no significant changes in G_Kur_ with the Courtemanche model, see Table 2). The results are consistent with the remodeling in those currents reported experimentally [36]–[38], and also highlights the importance of those conductances in the differences in AP between SR and cAF recordings. However, G_CaL_ increases in cAF with respect to SR with the three models, in contrast with previous studies showing a decrease in G_CaL_ [37]. The differences are likely to reflect the fact that I_CaL_ is both voltage and calcium dependent. Therefore, the effect of variability in G_CaL_ on the AP would also be dependent on calcium dynamics, which are different in the three baseline models.

**Table 2:** Percentage of variation of the median values subject to 95% confidence interval for G_K1_, G_NaK_, G_CaL_, G_to_, G_Kur_ and G_NaCa_ for SR and cAF with respect the default values in the Maleckar, Courtemanche and Grandi models, respectively.

	Maleckar		Courtemanche		Grandi	
	*SR*	*cAF*	*SR*	*cAF*	*SR*	*cAF*
**G_K1_**	−12±3%	+38±5%	+1±4%	+26±4%	+18±7%	+26±2%
**G_NaK_**	+27±7%	+51±5%	−2±5%	−10±5%	−27±3%	−45±1%
**G_CaL_**	+20±12%	+37±8%	+24±4%	+12±4%	+12±7%	+17±2%
**G_to_**	+16±15%	−10±9%	−23±5%	−35±5%	−2±6%	−10±2%
**G_Kur_**	+9±17%	−29±8%	−4±5%	−1±5%	−12±7%	−22±1%
**G_NaCa_**	+65±8%	+33±7%	+9±5%	+1±5%	+3±6%	+9±2%

Once the median value for each ionic conductance was estimated as described above, we refined the populations of sampled models by constructing newly generated populations of 2275 AP models with ionic currents sampled in the ±30% range around the estimated median physiological values. Both the sampling resolution and number of accepted models in the six final AP model populations was thus maximized, since most of the models accepted in the ±100% range population were within the ±30% range with respect to the median. This is also in good agreement with the ±30% range of variability considered in previous studies [7], [19], [39]. The refined human atrial model populations were then calibrated again to ensure that all models in the populations remained in range with their corresponding experimental ranges (SR or cAF) as described in Table 1.

The percentage of accepted models was 65.5% with the Maleckar model in SR and over 93% in the rest of cases, as shown in Table 3. The main cause of differences in the number of models excluded from the population is that the Maleckar model is more prone to generate DADs at slow pacing than the Courtemanche and Grandi models under certain electrophysiological conditions, such as reduced I_NaK_ current [26].

**Table 3:** Number and percentages of accepted sampled models in the experimentally-calibrated Maleckar, Courtemanche and Grandi populations in SR and cAF out of a total of 2275 sampled models.

	*SR*	*cAF*
Maleckar	1489 (65.5%)	2177 (95.7%)
Courtemanche	2275 (100%)	2271 (99.8%)
Grandi	2125 (93.4%)	2275 (100%)

### Statistical Analysis

Regression methods were used for the quantification of the main determinants of inter-subject electrophysiological variability [8], [13], [19]. The dependence of APD_20_, APD_50_ and APD_90_ on pairwise combinations of multiple ionic properties was analyzed using second-order multi-parametric regression on the accepted model populations. This was studied by calculating the coefficient of determination (R^2^) of the computed regressions: the higher R^2^ the stronger the dependence of APD on the considered parameters. A third parameter was included in the analysis for those cases where R^2^<0.7. Mechanisms of AP triangulation, calculated as the difference between APD_90_ and APD_50_, were also analyzed by regression techniques. The Mann-Whitney U test was used to determine statistical significance in differences on ionic conductance distributions between populations. Box plots, including median values and confidence intervals, as well as empirical cumulative distribution curves were constructed using the Matlab statistics toolbox (functions “boxplot” and “ecdf”, respectively).

## Results

### Ionic Determinants of Inter-subject APD Variability

**Figure 2:**
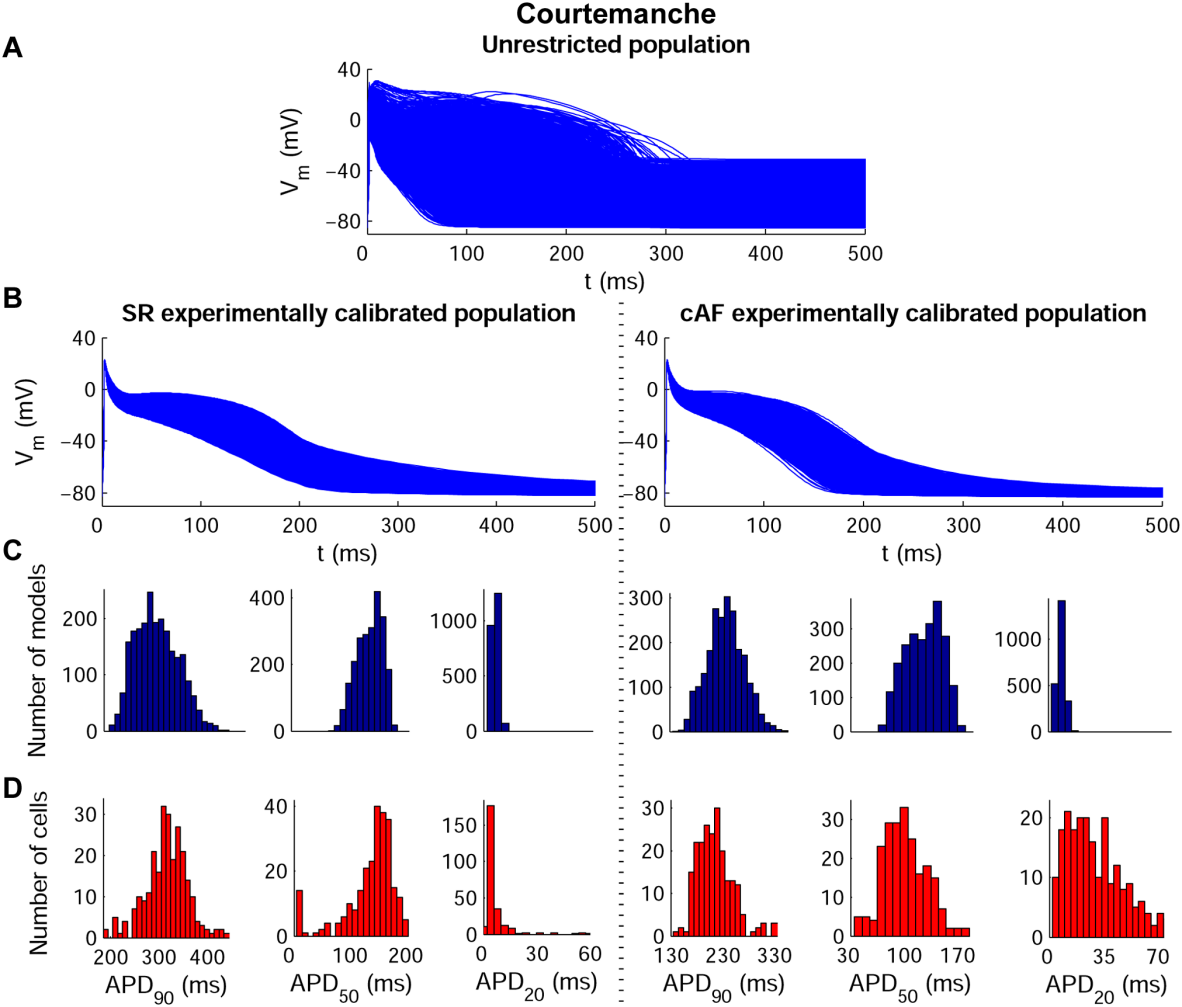
Experimentally-calibrated human AP model populations for SR and cAF based on the Courtemanche model. Initial unrestricted ±100% sampled population (**A**), experimentally calibrated ±30% sampled populations (**B**) and histograms corresponding to APD_90_, APD_50_ and APD_20_ distributions in both the calibrated model populations (**C**) and the experimental measurements (**D**). Histogram bar widths are 10 ms for both APD_90_ and APD_50_, and 4 ms for APD_20_.

**Figure 3:**
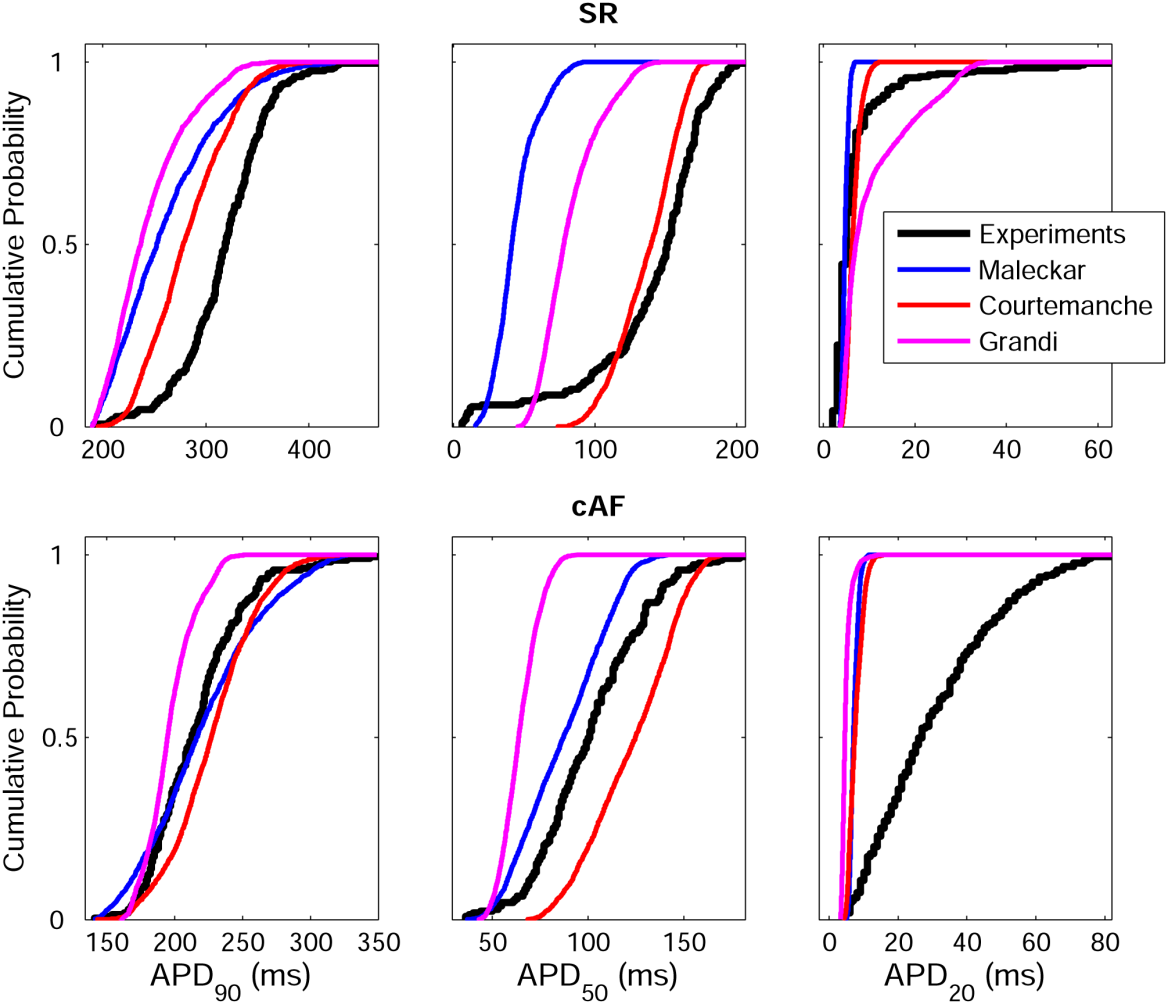
Empirical cumulative distribution curves for APD_90_, APD_50_ and APD_20_. Curves for experimental data (black) and the populations of models based on the Maleckar (blue), Courtemanche (red) and Grandi (magenta) models in both SR (top panels) and cAF (bottom panels).

**Figure 4:**
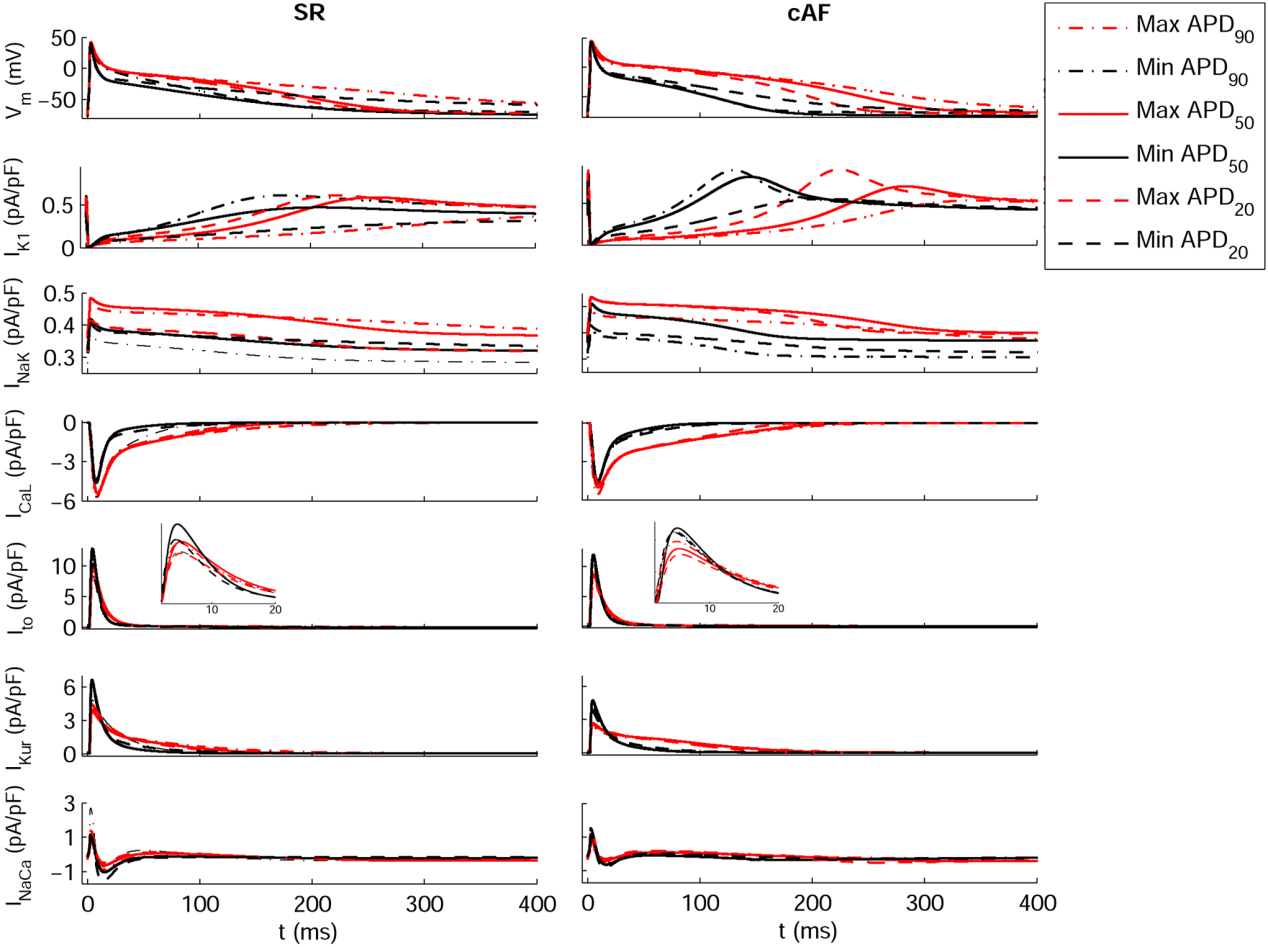
Transmembrane potential and ionic current traces in SR and cAF for the Maleckar model-based populations. Traces for models displaying maximum APD_90_ (red dash-dotted lines), minimum APD_90_ (black dash-dotted lines), maximum APD_50_ (red thin solid lines), minimum APD_50_ (black thin solid lines), maximum APD_20_ (red dashed lines) and minimum APD_20_ (black dashed lines). Insets provide detailed views of peak I_to_ current.

**Figure 5:**
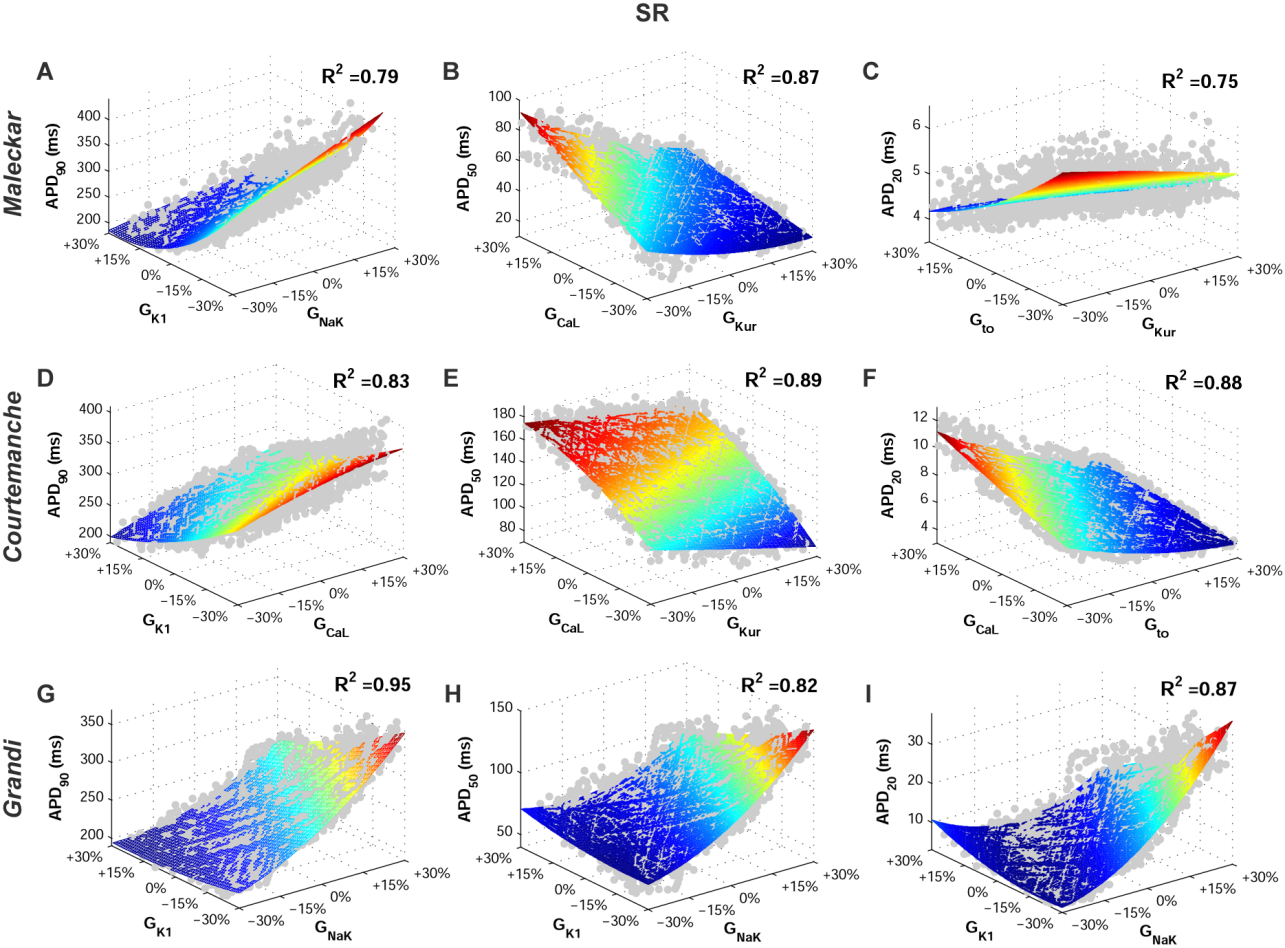
Ionic conductances determining inter-subject variability in human atrial repolarization in the SR AP model populations at 1 Hz. Regression surfaces for APD_90_, APD_50_ and APD_20_ are presented with respect to the two most significant ionic factors determining their variability, using populations based on the Maleckar (A–C), Courtemanche (D–F), and Grandi (G–I) models. Regression surfaces are color coded according to APD magnitudes, whereas each big dot denotes the value for one model in the calibrated populations.

**Figure 6:**
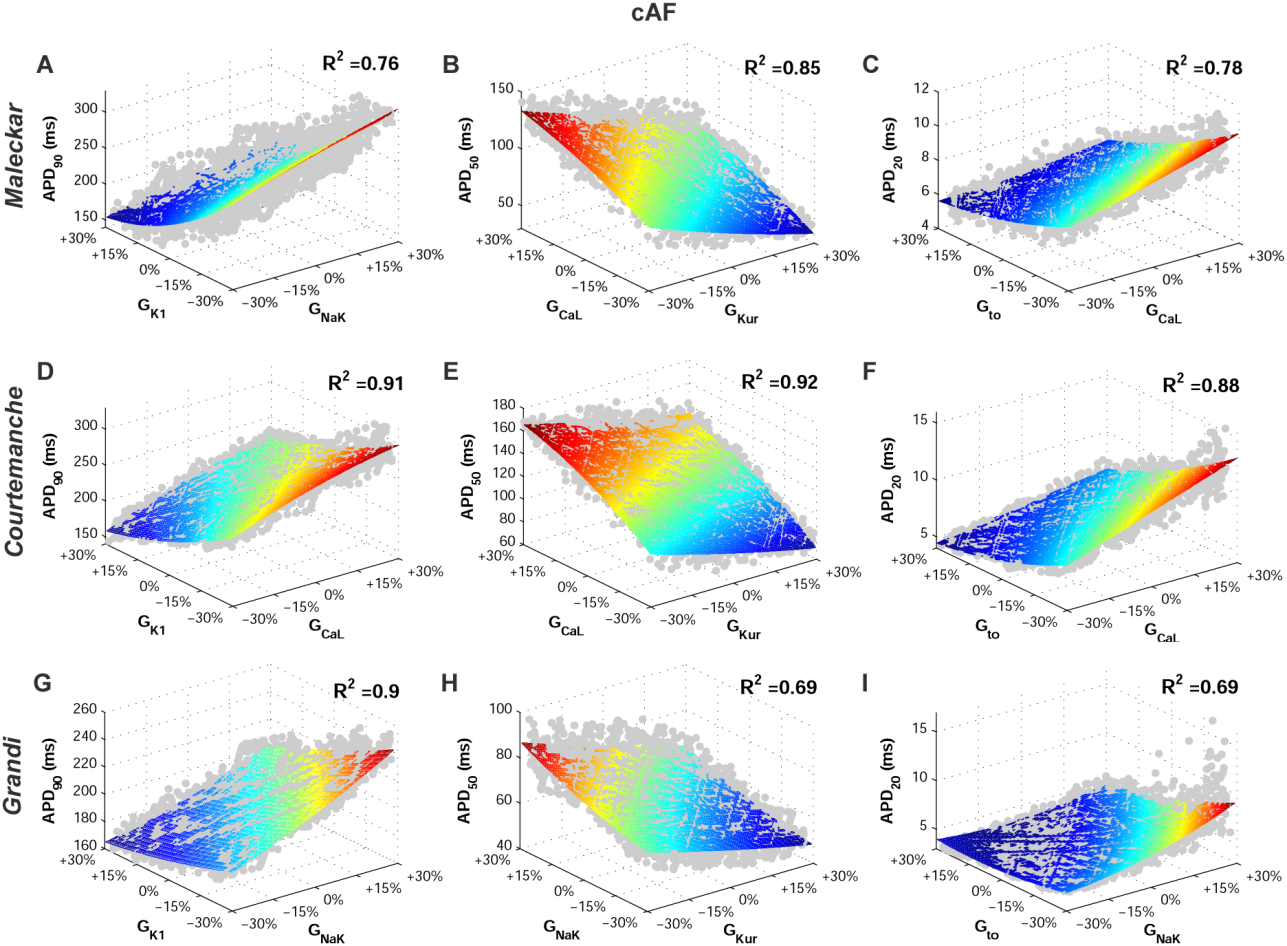
Ionic conductances determining inter-subject variability in human atrial repolarization in the cAF AP model populations at 1 Hz. Figure annotation as in Figure 5.

Figure 2 shows the wide range of AP morphologies for human atrial cell models in SR and cAF (left and right, respectively) obtained in simulations using the initial unrestricted population (with ionic currents sampled over a ±100% range of their original values, panel A), and the most restricted range of variability obtained with the experimentally-calibrated populations (panel B). Histograms show distributions of APD values obtained in the population in the simulations (panel C) and in the experimental recordings (panel D) across the experimental range. Results in Figure 2 are shown for the Courtemanche model and slightly different coverage of the experimental range is provided for the Maleckar and Grandi models (Figures S1 and S2 in Supplemental Material). In order to further illustrate the comparison between simulated and experimental APD distributions, Figure 3 shows the empirical cumulative distribution curves of the experimental and model population values of APD_90_, APD_50_ and APD_20_. The curves allow a clear comparison of the probability distributions and the degree of variability between experimental and simulated data. As illustrated in Figures 2, 3, S1 and S2, experimentally-calibrated populations of human atrial models are able to capture in most cases the wide inter-subject variability in AP duration and morphology exhibited in the experimental recordings, quantified using the properties and values shown in Table 1. The results also display differences between the populations generated with the 3 baseline models. In particular, the population based on the Courtemanche model covers similar ranges and with similar probability distributions to experimental APD_90_, APD_50_ and APD_20_ distributions in SR, but the agreement of APD ranges and probability distributions with experiments in cAF is not as good (Figures 2 and 3). In contrast, the population based on the Maleckar model provides the best agreement in the ranges and degrees of variability of APD_90_ and APD_50_ in cAF, but the agreement for APD values is worse in SR (Figure S1 and 3). The population with the Grandi model covers shorter ranges of APDs than experimental recordings, but the range and probability distribution of APD_20_ in SR is larger than those obtained with Courtemanche and Maleckar populations (Figure S2 and 3). Overall for all populations, the experimental range for APD_20_ in cAF is wider than the range obtained for simulated values for all three populations. This could be due to a variety of factors including the need for further refinement of the sampling methodology to allow for more parameters or wider parameter ranges to be considered, but it could also be due to the high sensitivity of APD_20_ to experimental protocols and conditions, which would require further investigations. Figure 4 illustrates the variability in the time-course of the ionic currents in the calibrated population with the Maleckar model and those underlying the maximum and minimum APD values measured at the different stages of repolarization in the human atrial models. Simulations show that most currents exhibit variability mainly in their peak value, as shown for I_to_, I_Kur_ and I_NaCa_. However, I_K1_, I_NaK_ and I_CaL_ exhibit inter-subject variability also in their sustained current densities. Particularly, the rate of decay of I_CaL_ was found to be markedly slower for cellular models with longer APD values, and more so in the cAF than in SR populations. Similar results were obtained with the Courtemanche, with slightly larger variability in I_Kur_ and I_NaCa_, and Grandi models, as shown in Figures S3 and S4, respectively. Figures 5 and 6 further illustrate the ionic mechanisms of inter-subject variability in the simulated human atrial AP models shown in Figure 4. They provide quantitative results on variability from the regression analysis described in the Methods for human atrial APD_90_, APD_50_ and APD_20_ with respect to their most important pairs of ionic modulators. Results are shown for the populations of sampled models constructed for the Maleckar (A–C), Courtemanche (D–F), and Grandi (G–I) in SR (Figure 5) and cAF conditions (Figure 6).

In spite of differences in ionic ranges between SR and cAF, simulations show similar ionic mechanisms of variability in SR and cAF (irrespective of the original underlying model). Variability in I_K1_ and importantly also in I_NaK_ entails variability in APD_90_ in both SR (Figures 5A, 5D and 5G) and cAF (Figures 6A, 6D and 6G). Results with the Courtemanche model population indicate an additional role of variability in I_CaL_ on variability in APD_90_ (Figure 5D; Figure 6D).

Regarding APD_50_ variability, our population-based analysis identifies variability in I_CaL_ and I_Kur_ as key at the early stage of repolarization, followed by a secondary role of variability in I_K1_ and I_NaK_, particularly with the Grandi model population, in both SR and cAF (Figures 5B, 5E and 5H; Figures 6B, 6E and 6H).

Finally, variability in APD_20_ strongly depends on variability in I_to_ and I_Kur_ in both the SR (Figures 5C and 5F) and cAF (Figures 6C, 6F and 6I) populations, as shown by comparing the results from all six model populations. These effects are concealed by changes in I_K1_ and I_NaK_ in the Grandi model population in both SR and cAF conditions (Figures 5I and 6I). Variability in I_CaL_ is relevant as well for the modulation of APD_20_ variability in the Courtemanche model population (Figure 4F).

**Figure 7:**
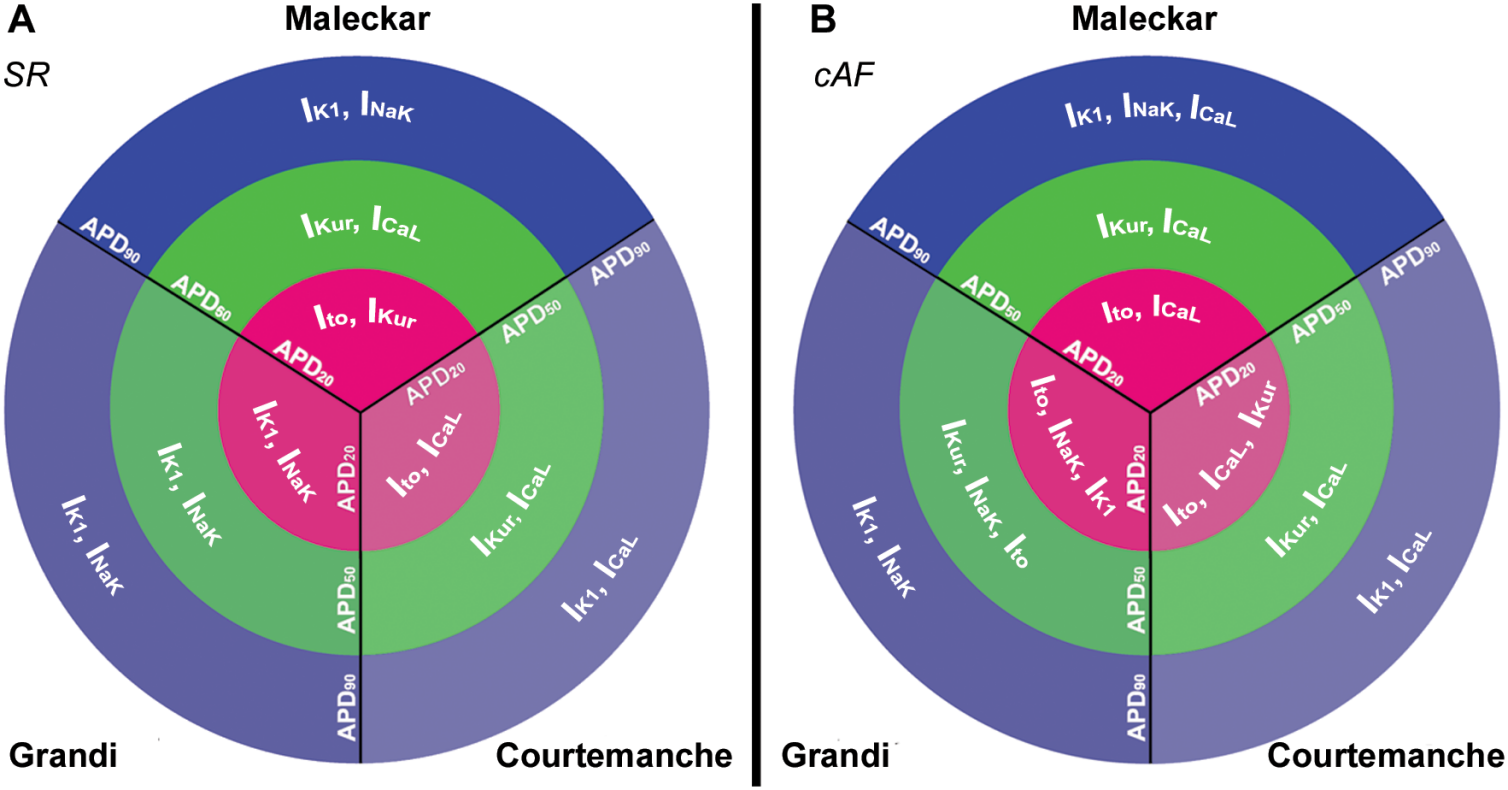
Ionic modulators of APD_90_, APD_50_ and APD_20_ in SR and cAF. Ionic conductances identified as having a stronger influence on human atrial cells APD_90_ (blue), APD_50_ (green) and APD_20_ (magenta), in SR (**A**) and cAF (**B**) with the Maleckar, Courtemanche and Grandi model populations.

Figure 7 summarizes the elucidated ionic mechanisms modulating APD in each repolarization stage with the model populations in SR (panel A) and cAF (panel B). In both SR and cAF, variability in I_K1_ and I_NaK_ modulate variability in APD_90_, I_CaL_ and I_Kur_ in APD_50_, and I_to_ and I_Kur_ in APD_20_. The main differences between SR and cAF in terms of ionic mechanisms are found in the populations based on the Grandi model, which depict decreased relevance of variability in I_K1_ and I_NaK_ in modulating the early stages of atrial cell repolarization in the cAF Grandi models, and a reduced role of variability in I_CaL_ in regulating APD at all stages of repolarization in cAF.

### Ionic Determinants of Variability in AP Morphology

**Figure 8:**
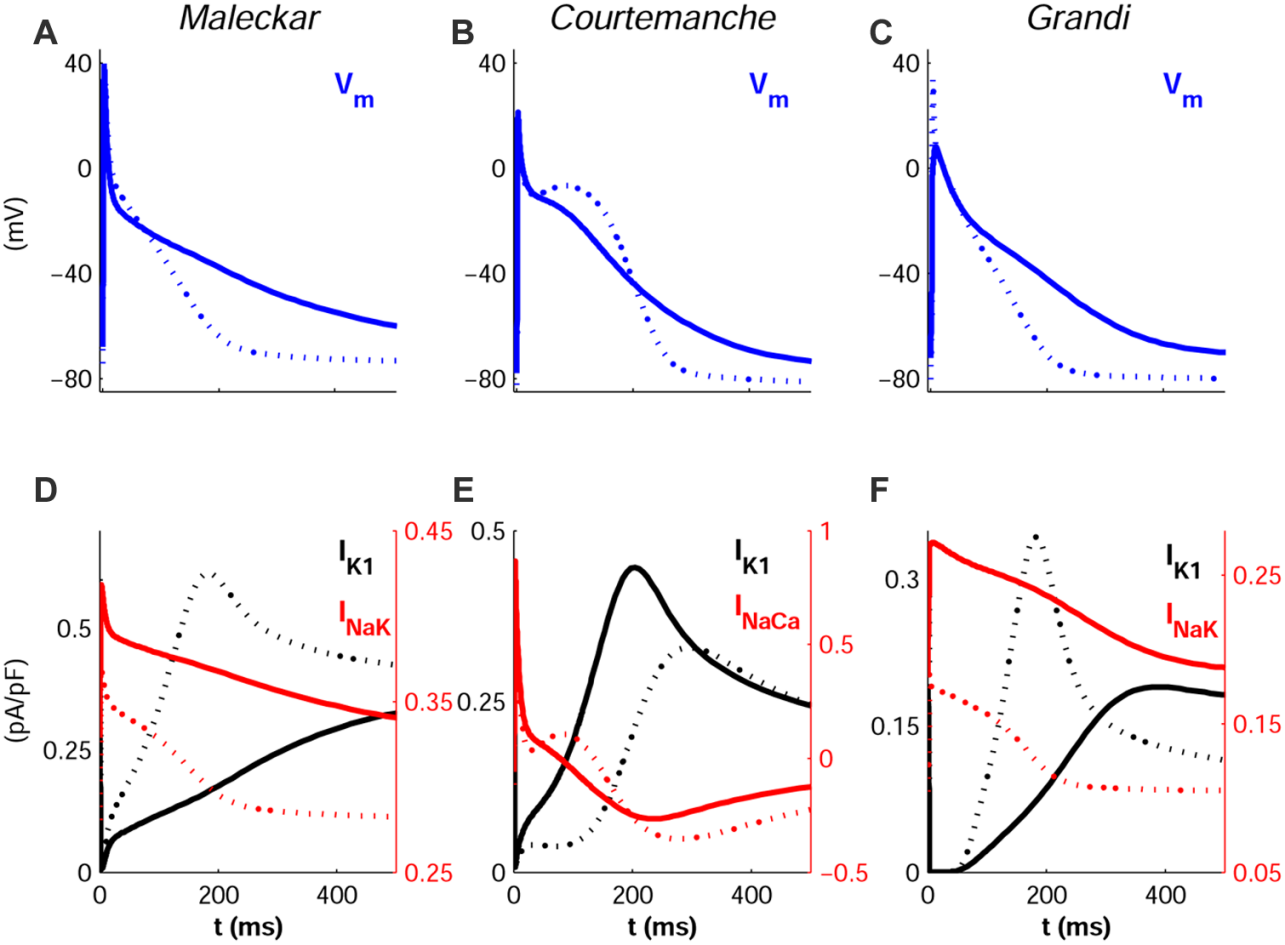
Inter-subject variability in human atrial AP triangulation. The most and least triangular APs in each population are shown (solid and dotted lines, respectively), obtained with the Maleckar (**A**), Courtemanche (**B**) and Grandi (**C**) models in SR. Corresponding time-course of ionic mechanisms of AP triangulation are shown: I_K1_ and I_NaK_ (**D** and **F**), I_K1_ and I_NaCa_ (**E**).

Figure 8 shows the most significant differences in AP morphology, quantified through AP triangulation, obtained within the populations based on the Maleckar (Figure 8A), Courtemanche (Figure 8B) and Grandi (Figure 8C) models. Our simulation results show that our populations of models are able to capture a large inter-subject variability in AP morphology, as measured in our recordings and also reported in previous studies [40]–[42]. The regression analysis reveals variability in I_K1_ and I_NaK_ as the main underlying mechanisms of AP triangulation in the Maleckar and Grandi model populations (R^2^>0.96), whereas the combination of changes in I_K1_ and I_NaCa_ leads to the highest regression values with the Courtemanche model populations (R^2^ = 0.86). Figure 8 (panels D and F) further identifies the combination of I_K1_ with either I_NaK_ or I_NaCa_ (depending on the model) as the main properties leading to the different AP morphologies. The most triangular APs were generated for combinations of decreased I_K1_ and increased I_NaK_ (Maleckar and Grandi) or increased reverse-mode of the I_NaCa_ (Courtemanche) current amplitudes.

### Inter-subject Variability in Rate Dependence

Inter-subject variability in APD rate dependent properties of human atrial cardiomyocytes was also investigated by comparing the response of our populations of sampled human atrial models at 1 Hz, 2.5 Hz and 3 Hz pacing rates. Overall, our results are qualitatively similar for the three pacing rates and the role played by the ionic currents at the different stages of repolarization is similar at the three pacing rates. Only an increased relevance of variability in I_CaL_ and I_Kur_ on the last repolarization stage can be found at fast pacing. Pacing rates faster than 3 Hz were not studied since they result in the stimulus application during the repolarization phase of the cellular models with longer APD_90_ within the populations. This would lead to stimulus-induced APD prolongation, and would create artefacts in the values of APD measured.

**Figure 9:**
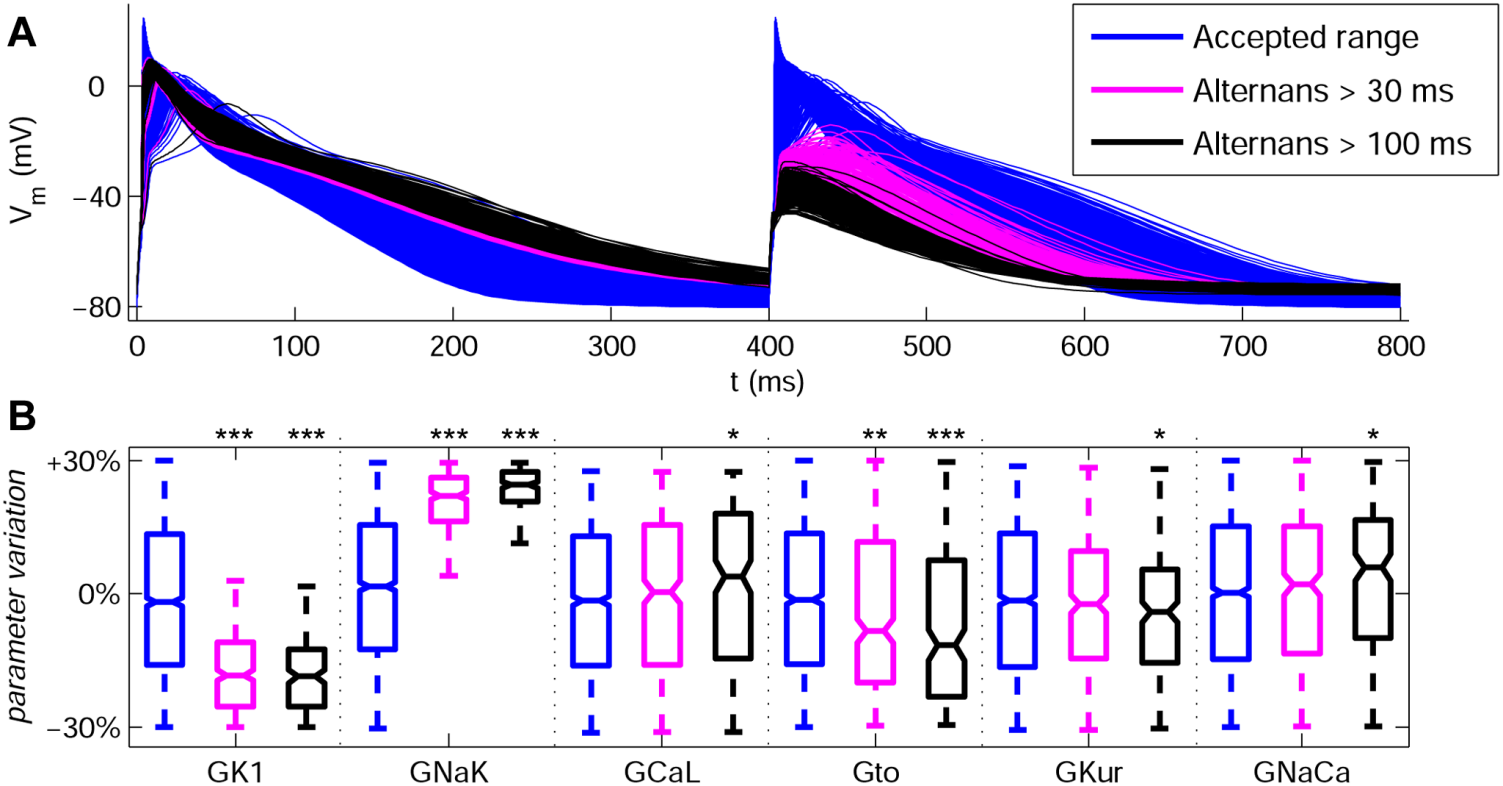
AP traces and variability of ionic conductances with the population based on the Grandi model in SR at 2.5 Hz. (**A**) Accepted models as for 1 Hz pacing are shown in blue, whereas models exhibiting pronounced AP alternans (|APD_90,odd_ – APD_90,even_| >30 ms) and strong AP alternans (|APD_90,odd_ – APD_90,even_| >100 ms) are shown in magenta and black, respectively. (**B**) Variability of ionic conductances G_K1_, G_NaK_, G_CaL_, G_to_, G_Kur_ and G_NaCa_ for the models in panel **A**. Each boxplot represents the range covered by the ionic conductances: the edges of the box are the 1^st^ and 3^rd^ quartiles, the whiskers extend to the most extreme datapoints, the estimated median physiological value is the central horizontal line and the notch around the median is the 5% significance level. (Mann-Whitney U test vs. accepted range: *p<0.05; **p<0.01; ***p<0.001).

A specific feature of the Grandi population is that the models are particularly prone to exhibit AP alternans at fast pacing rates, with low amplitude and slow upstrokes in a significant percentage of the models within both SR and cAF populations. This is in agreement with previous experimental studies [43]–[46]. Figure 9 (panel A) shows clear alternating behavior in a large number of models in the Grandi model population in SR for 2.5 Hz pacing. The alternating behavior occurs in models with both low amplitude of I_K1_ and increased activity of I_NaK_ (Figure 9B). This is in good agreement with the important role played by both ionic currents in modulating APD at the different stages of repolarization (Figure 7). Furthermore, Figure 9 (panel B) shows the effects of the rest of ionic currents in generating alternans are less statistically significant and I_to_ is the only additional ionic current with a notable effect in its variability.

## Discussion

In this study, populations of models based on three human atrial AP models are able to mimic a wide range of inter-subject variability in human atrial AP properties as exhibited in a comprehensive set of electrophysiological recordings, obtained from over 350 SR and cAF patients [6]. In our analysis, we considered recordings obtained in a large number of patients with different etiologies, and therefore exhibiting a remarkable range of APD_90_ variability of up to 250 ms in SR and 190 ms in cAF. Our results show that a large range of the experimental AP variability can be recovered in the simulations with the model populations. Relatively modest ±30% variations in ionic conductances are often sufficient using the three models, and around median values that also deviate less than 30% from their original values. We specifically consider three widely used different human atrial AP models to assess potential differences and similarities in the results obtained with them.

The actual “true” range of ionic variability in the specific experimental samples used to record the APs is of course unknown. The AP traces do not contain enough information to uniquely determine all ionic properties. Furthermore, ionic currents cannot be measured in tissue, and voltage clamp measurements have the inherent limitations of using isolated single cells, whose channels are known to be affected by the isolation procedure [47]. In fact, even if we were able to measure the ionic conductances at a specific moment in time, they are subject to continuous variations caused by extrinsic factors, such as circadian rhythms in plasma level concentrations [4] or long term drug effects [5]. Therefore, through our computational populations of models approach, we aim at and are able to identify and suggest what may be likely ranges and important players in explaining the variability in the human AP recordings [48]–[50]. This is no different to any other theoretical or experimental modelling study, which should aim at probing and refining our understanding of biological systems [51], [52]. The credibility of our findings is supported by similarities in the mechanisms identified using the three different models, and also by their agreement with previous experimental and theoretical studies. Further studies will aim at challenging our predictions and methodologies under different clinical and experimental conditions.

Through the combination of our population approach with a large experimental recordings dataset, we therefore expand our understanding of potential underlying causes of human atrial AP variability. An important methodological novelty is that it allows identifying how synergistic combinations of various ionic current densities could determine inter-subject variability in the human atrial AP, which goes an important step beyond previous sensitivity analysis methods [7], [39], [53]. Therefore, we are able to suggest how complex non-linear combinations of simultaneous variability in multiple ionic conductances, as may be present in different individuals, lead to differences in atrial cellular repolarization in SR versus cAF models. Our results may provide the basis for a deeper understanding on the penetration of different pharmacological therapies at the population level, which is critical in the interpretation of outcomes for anti-arrhythmic drug development and the lack of pharmacological response in some individuals. This could be the focus of further studies, as was done in [9] for dofetilide in rabbit Purkinje studies.

The median electrophysiological values and ranges extracted from the initial populations differed between SR and cAF, as shown in Figure 1. Calibration of the populations with cAF recordings leads to notably higher median values of G_K1_ in cAF, reduction in G_to_ and reduction in G_Kur_ (the latter to a lesser extent in the Courtemanche model). The predictions in repolarizing currents are in good agreement with the ionic remodeling observed in cAF atrial cardiomyocytes in previous experimental studies [36]–[38].

The analysis of the six model populations yields in most cases consistent results in terms of the ionic properties identified as determining variability in the different phases of the AP repolarization. Variability in I_K1_ and I_NaK_ is identified as key in explaining inter-subject variability in APD_90_ and AP morphology. Our results therefore support the well-established importance of I_K1_ in human atrial electrophysiology [36], [54]. Alterations in I_K1_ may also modulate variations in APD_50_ and APD_20_ by modifying cellular excitability through the resting membrane potential.

Importantly, our study also highlights the importance of variability in I_NaK_ in inter-subject variability in APD_90_, which further supports the results of our previous study [34]. Our findings suggest the need for pharmacological assessment of potential drug effects on the sodium/potassium pump (as is the case of amiodarone, for example) due to its importance on atrial repolarization, in addition to effects on currently evaluated currents such as sodium, potassium and calcium channels [55]–[58].

Our population-based results also highlight the importance of variability in I_CaL_ in determining inter-subject variability in APD_50_. However, its role in modulating variability in APD_90_ and APD_20_ is less significant. This may explain why some calcium channel blockers, such as verapamil, significantly reduce the degree of electrical remodeling, but only yield a minimal reduction in inducibility of AF, despite aiming to modify tissue refractoriness in AF patients [59].

Due to its atria specificity and negligible ventricular expression levels, I_Kur_ has been previously proposed as a potentially-important ionic target for atrial antiarrhythmic therapies aiming at exclusively prolonging atrial refractoriness [20], [42], [60]–[64]. Our results support its importance in modulating inter-subject variability in APD_50_ and APD_20_, with smaller importance in modulating APD_90_. Another important modulator of variability in APD_20_ is variability in I_to_, which in contrast has only small effects on APD_90_ and APD_50_. This supports the potential of drugs such as AVE0118, which interfere with both I_to_ and I_Kur_, and have been shown to modulate atrial repolarization with no apparent effects on ventricular repolarization [20], [25], [61], [62]. Further quantitative studies using the population-based approach could be conducted to investigate the implications of variability in G_to_ and G_Kur_ in modulating the response to pharmacological block in SR and cAF.

Finally, our results suggest a secondary role of I_NaCa_ in modulating inter-subject APD variability, as its effects are less prominent than those of the other currents. However, its influence on AP morphology cannot be neglected. Recent I_NaCa_ inhibitors have shown the potential to prevent arrhythmogenic events in ventricular myocardium by decreasing the amplitude of pharmacologically-induced early and delayed afterdepolarizations [65], [66], although their effects in atrial tissue still remain unexplored.

The relative importance of certain ionic currents in the modulation of APD variability displays differences between the populations based on the three AP models. In particular, the relevance of I_NaK_ in modulating variability in APD_90_ with the Courtemanche model population is high but masked by the even bigger relevance of I_K1_ and I_CaL_. Similarly, the relative importance of I_Kur_ in modulating APD_20_ is masked by those of I_to_ and I_CaL_ with the Courtemanche model population, and by I_K1_ and I_NaK_ with the Grandi model population.

As reviewed in previous papers [6], [26]–[28] and briefly summarized in the Methods section, the three human atrial models display differences both in equations and parameter values in the formulations of transmembrane currents and calcium dynamics. In this study, through the population-based approach, we are able to assess the relative importance of conductance values versus model structure in explaining differences in model outputs, and specifically we are able to identify similarities and differences in the ability of the populations based on the three AP models to reproduce experimental APD ranges when conductances are varied. Our study highlights that, whereas the experimentally-reported variability in APD_90_ and APD_50_ is largely reproduced by the populations in SR and cAF, the large experimental variability in APD_20_ in cAF is difficult to capture and none of model populations covers its full range, as shown in Figures 2 and 3. Furthermore, the population based on the Courtemanche model for instance provides the best agreement in terms of distributions of APD in SR with respect to those reported experimentally, whereas the population based on the Maleckar model provides a better agreement with experimental APD data in cAF, probably due to its triangular shape. The population based on the Grandi model in contrast seems better suited for studies related to, for example, the AP upstroke, since it shows higher variability than with the Courtemanche and Maleckar models populations, or mechanisms of arrhythmia due to its ability to reproduce alternating behavior at fast pacing rates. The differences in the simulated APs obtained based on the three original models may arise from differences in the transmembrane current formulation but also importantly through differences in the calcium dynamics, as highlighted in previous studies [26], [27].

### Limitations

The populations of human atrial models developed in the present study are based on a large amount of experimental recordings obtained from trabeculae extracted from the right atrial appendage, which is available from biopsies. Cardiomyocytes from other atrial locations may exhibit a different degree of variability in AP [67], which could be investigated using a similar approach to the one proposed in our study. In this paper, populations of sampled models account for inter-subject variability in the action potential of human atrial cells, which in tissue preparations may be affected by inter-cellular coupling. Effects of tissue coupling could be investigated in further studies, aiming at translating the conclusions obtained from cellular to tissue model populations. Furthermore, experimental data at faster pacing rates than 1 Hz could help in the populations calibration and the elucidation of the ionic mechanisms underlying AP variability in rate dependence [13].

In our study, we examined the effects of variability in a subset of conductances, which were chosen based on their importance in determining the atrial AP using a sensitivity analysis [34]. It is however possible that other electrophysiological properties, such as ionic concentrations or calcium handling, and additional ionic currents, such as the background chloride current (I_bCL_), may play a role in APD variability. These additional important factors could be the focus of future investigations.

## Conclusions

Our study shows the ability of populations of human atrial cell models to mimic the remarkable inter-subject variability in human atrial AP duration and morphology measured in over 450 biopsy samples obtained from SR and cAF patients. Three different human atrial cell models are used to construct the populations of human atrial cell models in SR and cAF, in order to analyse and evaluate similarities and differences between them. Our simulation results reveal that relatively modest variations in ionic currents of ±30% with respect to their original values yield APD ranges of 250 ms in SR and 190 ms in cAF in the model populations.

The main ionic mechanisms modulating inter-subject variability in the different phases of the AP are very similar in SR and cAF populations using the three baseline models. In all cases, I_CaL_, I_to_ and I_Kur_ are key in modulating inter-subject differences in APD_20_ and APD_50_, whereas I_K1_ and I_NaK_ determine patient-specific values of APD_90_. Elucidating likely mechanisms underlying inter-subject variability in atrial electrophysiological properties may be crucial in the understanding of inter-subject differences in human atrial dynamics and the response to anti-AF pharmacological therapies. The fact that similar ionic mechanisms are reported using different models lends credibility to our findings.

## Supporting Information

Experimental and simulation data accompanying this publication are available for download at: http://dx.doi.org/10.6084/m9.figshare.1031569.

## Supporting Information

Figure S1 **Experimentally-calibrated human AP model populations for SR and cAF based on the Maleckar model.** Initial unrestricted ±100% sampled population (**A**), experimentally calibrated ±30% sampled populations (**B**) and histograms corresponding to APD_90_, APD_50_ and APD_20_ distributions in both the calibrated model populations (**C**) and the experimental measurements (**D**). Histogram bar widths are 10 ms for both APD_90_ and APD_50_, and 4 ms for APD_20_.
(TIF)

Figure S2 **Experimentally-calibrated human AP model populations for SR and cAF based on the Grandi model.** Initial unrestricted ±100% sampled population (**A**), experimentally calibrated ±30% sampled populations (**B**) and histograms corresponding to APD_90_, APD_50_ and APD_20_ distributions in both the calibrated model populations (**C**) and the experimental measurements (**D**). Histogram bar widths are 10 ms for both APD_90_ and APD_50_, and 4 ms for APD_20_.
(TIF)

Figure S3 **Transmembrane potential and ionic current traces in SR and cAF for the populations based on the Courtemanche model.** Traces in SR (left) and cAF (right) for models displaying maximum APD_90_ (red dash-dotted lines), minimum APD_90_ (black dash-dotted lines), maximum APD_50_ (red thin solid lines), minimum APD_50_ (black thin solid lines), maximum APD_20_ (red dashed lines) and minimum APD_20_ (black dashed lines).
(TIF)

Figure S4 **Transmembrane potential and ionic current traces in SR and cAF for the populations based on the Grandi model.** Traces in SR (left) and cAF (right) for models displaying maximum APD_90_ (red dash-dotted lines), minimum APD_90_ (black dash-dotted lines), maximum APD_50_ (red thin solid lines), minimum APD_50_ (black thin solid lines), maximum APD_20_ (red dashed lines) and minimum APD_20_ (black dashed lines).
(TIF)
