# Supplementary figures and images for: Inter-Subject Variability in Human Atrial Action Potential in Sinus Rhythm versus Chronic Atrial Fibrillation
Source: PLoS One. 2014 Aug 26;9(8):e105897. doi: 10.1371/journal.pone.0105897 (PMC4144914; doi:10.1371/journal.pone.0105897)

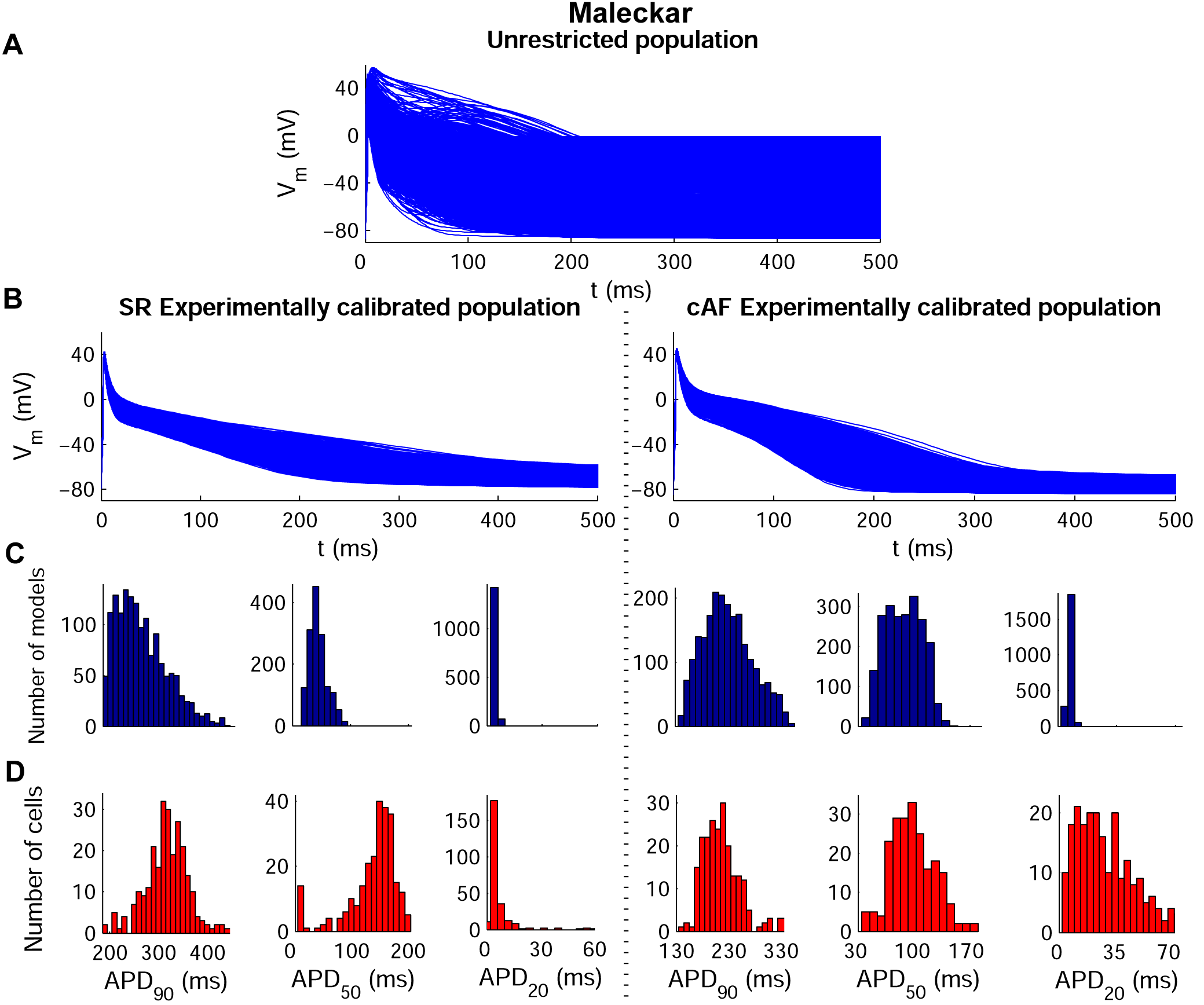

Supplement: Figure S1 — Experimentally-calibrated human AP model populations for SR and cAF based on the Maleckar model. Initial unrestricted ±100% sampled population (A), experimentally calibrated ±30% sampled populations (B) and histograms corresponding to APD90, APD50 and APD20 distributions in both the calibrated model populations (C) and the experimental measurements (D). Histogram bar widths are 10 ms for both APD90 and APD50, and 4 ms for APD20. (TIF) [file pone.0105897.s001.tif]

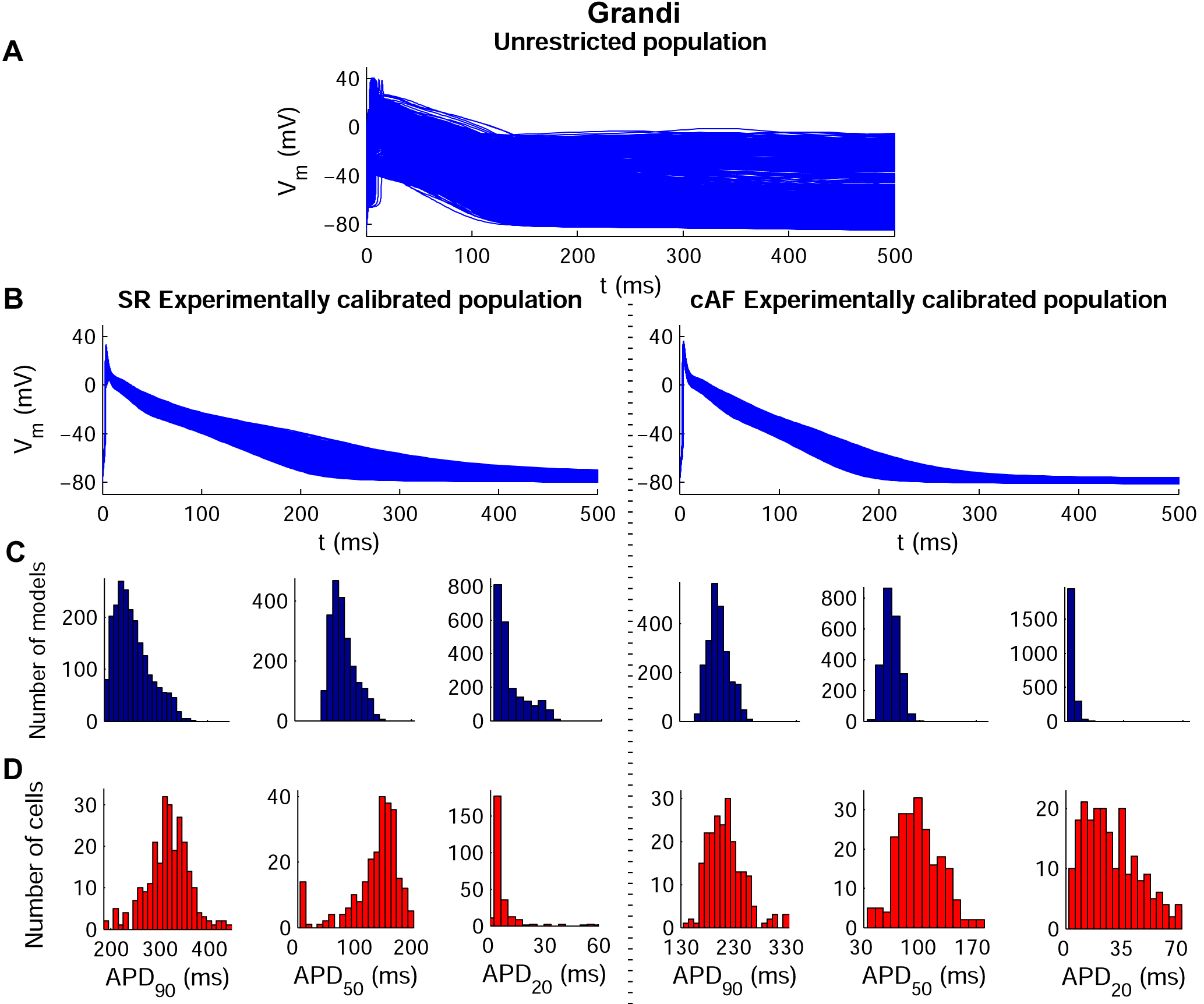

Supplement: Figure S2 — Experimentally-calibrated human AP model populations for SR and cAF based on the Grandi model. Initial unrestricted ±100% sampled population (A), experimentally calibrated ±30% sampled populations (B) and histograms corresponding to APD90, APD50 and APD20 distributions in both the calibrated model populations (C) and the experimental measurements (D). Histogram bar widths are 10 ms for both APD90 and APD50, and 4 ms for APD20. (TIF) [file pone.0105897.s002.tif]

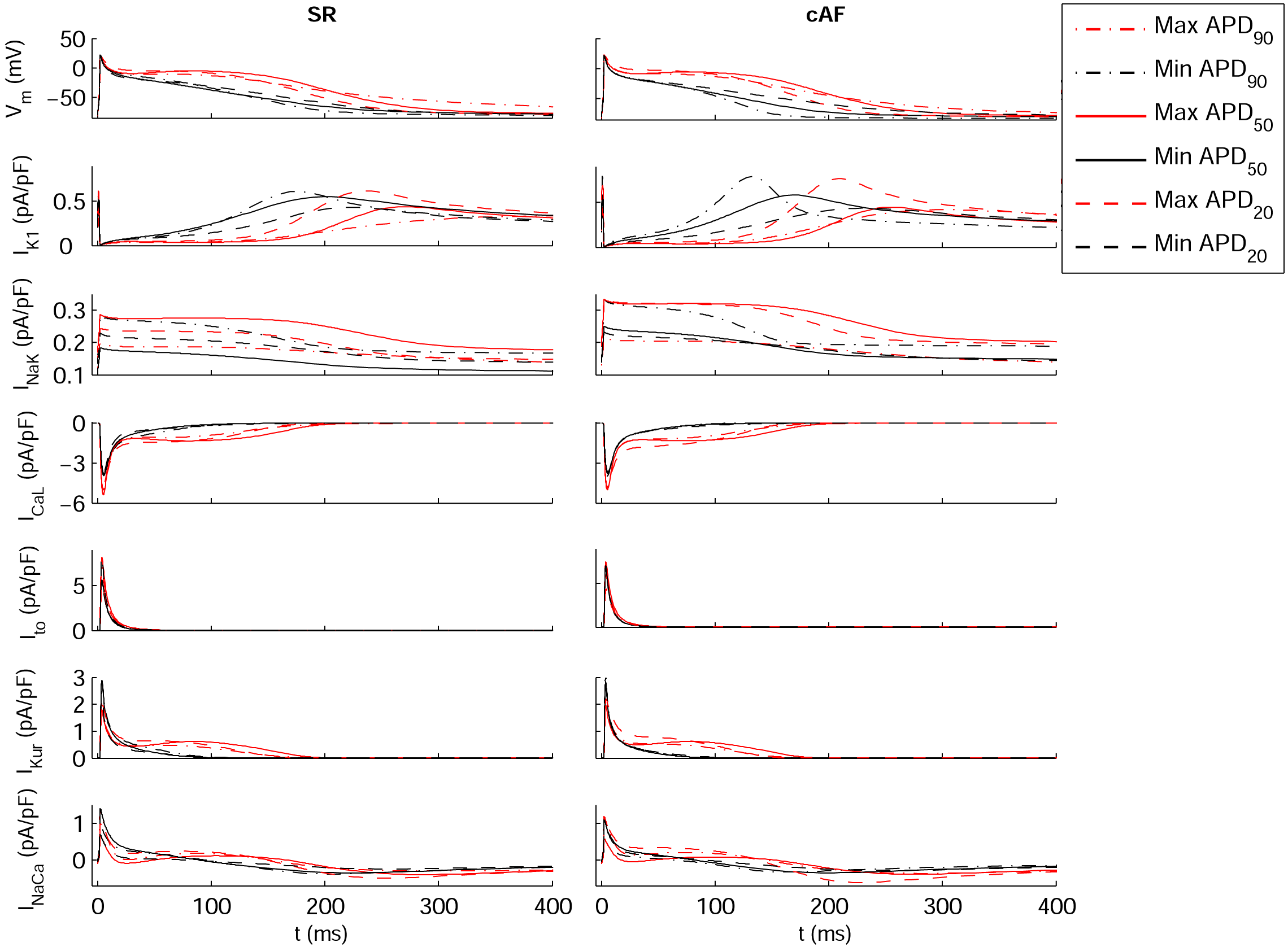

Supplement: Figure S3 — Transmembrane potential and ionic current traces in SR and cAF for the populations based on the Courtemanche model. Traces in SR (left) and cAF (right) for models displaying maximum APD90 (red dash-dotted lines), minimum APD90 (black dash-dotted lines), maximum APD50 (red thin solid lines), minimum APD50 (black thin solid lines), maximum APD20 (red dashed lines) and minimum APD20 (black dashed lines). (TIF) [file pone.0105897.s003.tif]

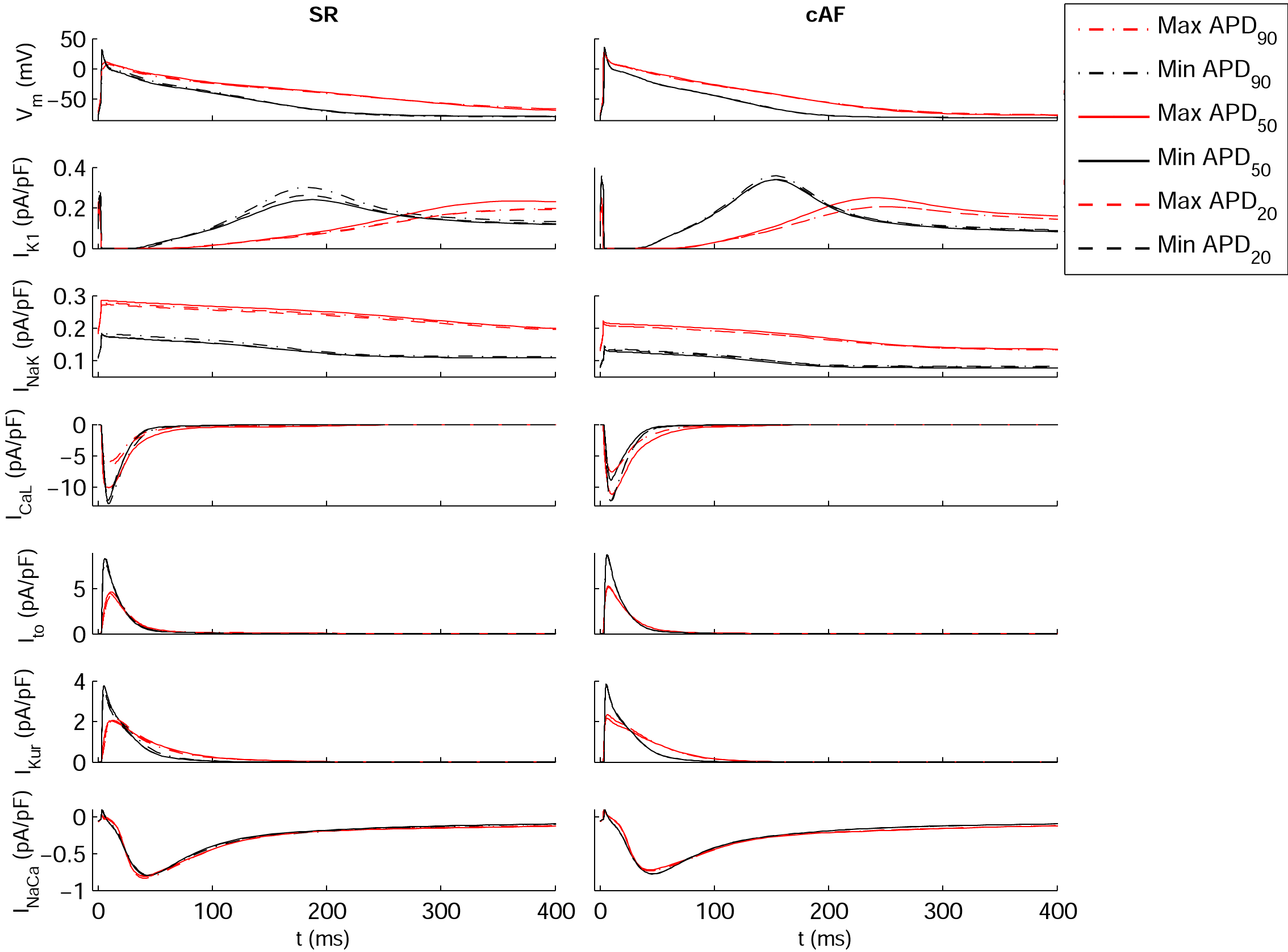

Supplement: Figure S4 — Transmembrane potential and ionic current traces in SR and cAF for the populations based on the Grandi model. Traces in SR (left) and cAF (right) for models displaying maximum APD90 (red dash-dotted lines), minimum APD90 (black dash-dotted lines), maximum APD50 (red thin solid lines), minimum APD50 (black thin solid lines), maximum APD20 (red dashed lines) and minimum APD20 (black dashed lines). (TIF) [file pone.0105897.s004.tif]
